# A Newly Dated Late Pleistocene and Holocene Archaeological Assemblage from Bukhtarma Cave in the Southern Altai Piedmont, East Kazakhstan

**DOI:** 10.1007/s41982-024-00187-x

**Published:** 2024-07-16

**Authors:** Radu Iovita, William Rendu, Susanne Lindauer, Zhaken Taimagambetov, Galina A. Kushch, Gennady F. Baryshnikov

**Affiliations:** 1 Department of Geosciences, University of Tübingen, Tübingen, Germany; 2 Center for the Study of Human Origins, Department of Anthropology, New York University, New York, NY USA; 3 ZooStan - ArchaeoZoological Center for the study of Central Asia - CNRS – Al-Farabi Kazakh National University – International Research Laboratory, Almaty, Kazakhstan; 4 Curt-Engelhorn-Zentrum Archäometrie, Mannheim, Germany; 5 National Museum of the Republic of Kazakhstan, Astana, Kazakhstan; 6 East Kazakhstan Regional Museum of Local History, Öskemen, Kazakhstan; 7 Laboratory of Theriology, Zoological Institute of the Russian Academy of Sciences, St. Petersburg, Russian Federation

**Keywords:** Central Asia, Altai, Middle Paleolithic, Upper Paleolithic, Neolithic, Bronze Age, Zooarchaeology, Lithics

## Abstract

The Altai mountains contain a number of cave and rockshelter sites that have given crucial information about human evolution in Asia. Most of these caves are located in the Gornyi Altai of Siberia, while the southern flank of the range remains much less known. Bukhtarma Cave was a karstic cave located near the former village of Peshchera, on the banks of the Bukhtarma River running through the foothills of the southern (Kazakh) Altai mountains. The Soviet East Kazakhstan Archaeological Expedition carried several excavation campaigns in the cave in the early to mid 1950s, discovering Paleolithic stone tools as well as animal bones. The collections were split between the East Kazakhstan Regional Museum of Local History in Öskemen (the lithic and part of the faunal collection) and the Zoological Institute in Leningrad (now St. Petersburg) (most of the fauna). Subsequently, the site was flooded by the construction of the Bukhtarma Reservoir in 1958, such that further fieldwork is impossible. However, in 2020, we reanalyzed the zooarchaeological collections and obtained several ^14^C dates. Based on the excavation documentation and the newly obtained dates exclusively taken from cut marked and carnivore-modified bone, we reconstruct at least three Paleolithic archaeological horizons, spanning the time between ca. 47–30 ka cal BP and exhibiting Middle and Upper Paleolithic characteristics, as well as the remains of several Holocene occupations, the latest of which dates to the Bronze Age. We present here a summary of the lithic and faunal assemblage and draw general conclusions about the site’s placement within the regional Paleolithic.

## Introduction

**Fig. 1:**
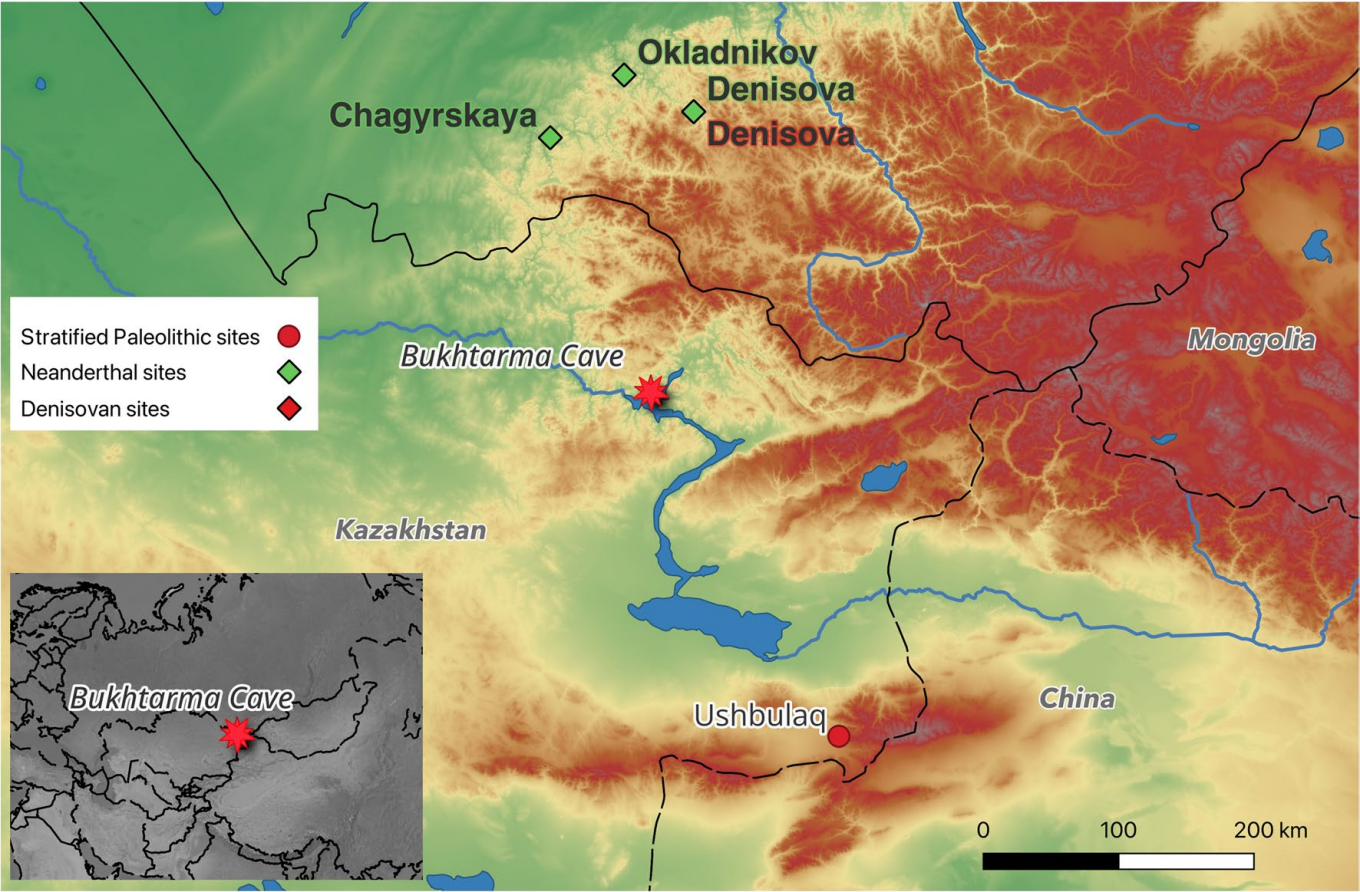
Map showing the location of Bukhtarma Cave and nearby important Paleolithic sites

Considerable new efforts to discover new Paleolithic sites in Central Asia have been made in the last decade (e.g., Derevianko et al., 2017; Iovita et al., 2020; Krajcarz et al., 2016; Kunitake & Taimagambetov, 2021; Ozherelyev et al, 2023a, b; Pavlenok et al., 2021; Rybin et al., 2014). Several projects, including our own, have now surveyed the area located in Kazakhstan immediately south of the archaeologically rich Gorny Altai (Derevianko et al., 1998), but no significant new sites have been found. Therefore, revisiting old sites that have already been studied by Soviet scholars is of paramount importance. Unfortunately, most of these lay along the Irtysh and Bukhtarma rivers in East Kazakhstan (Krylova, 1959; Taimagambetov & Ozherelyev, 2009), and were flooded due to the construction of the Bukhtarma Reservoir in 1958 and many have been submerged. Because most of them were open-air sites with poor organic preservation, there is no way to reconstruct their chronology from museum collections. Bukhtarma Cave, the first documented Paleolithic cave site in Kazakhstan, constitutes a notable exception to this rule, because its faunal collections still exist. In this report, we provide a maximal possible reconstruction of the site using the available data (Fig. 1).

## Regional Setting and General Characteristics

**Fig. 2:**
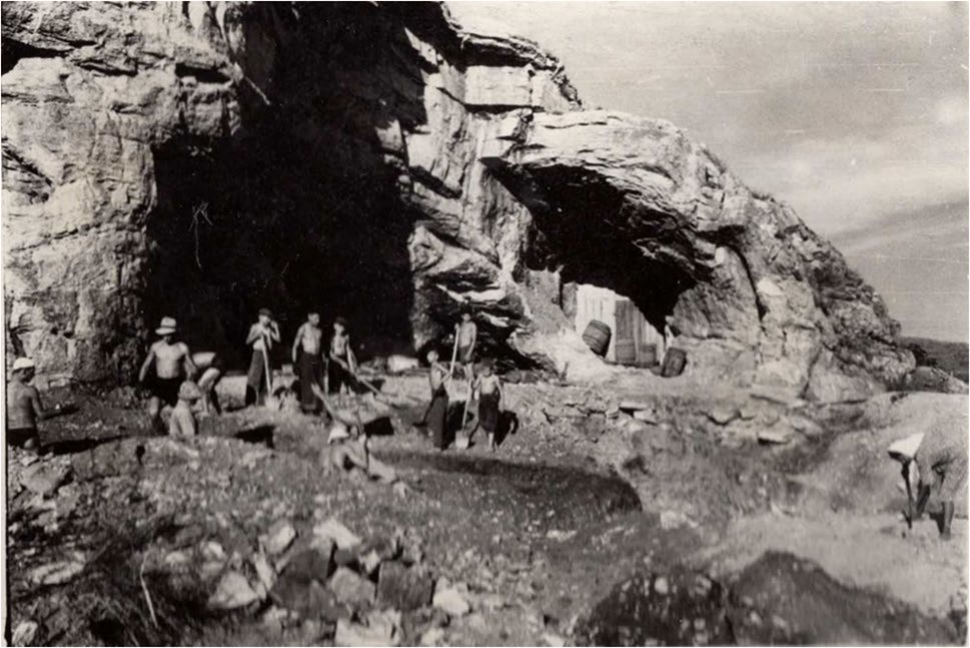
Photograph of the 1953 excavation (courtesy of the East Kazakhstan Regional Museum of Local History, Öskemen, Kazakhstan)

The cave was located on the right bank of the Bukhtarma River, a tributary of the Irtysh carving the Carboniferous limestone foothills of the southern Altai in East Kazakhstan. It was first described by Spassky (1818) and then excavated as part of the Hermitage’s East Kazakhstan Archaeological Expedition (Chernikov, 1952; Gokhman, 1957). The site was originally published as Пeщepa/Peshchera, which simply means “cave” in Russian, and could lead to confusion with many other cave sites. We therefore redubbed it here using the river’s English name (in Russian Бyxтapминcкaя or Bukhtarminskaya/Bukhtarminskai͡a (cf. Panteleev, 2015); in Kazakh (Cyrillic) Бұқтыpмa/(Latin): Buqtyrma Üñgırı)). According to Gokhman’s (1957) description, the cave was situated to the east of the now submerged eponymous village of Peshchera, about 1.5 km from the modern river channel and ca. 30 m above it (Fig. 2).

Probably, the river flowed closer to the cave during prehistoric times, because Gokhman notes that a test pit 16 m from the entrance contained river cobbles found at ~ 9 m below the cave surface (Gokhman, 1957:54). The cave entrance faced west and consisted of two chambers, named “Big” and “Small,” united by a common, partially collapsed roof (see Fig. 1). The East Kazakhstan Expedition (Chernikov, 1952) began excavating in 1950 and continued until 1954. A total surface of 130 m^2^ was excavated in both chambers. A test pit laid in the larger chamber yielded no finds so we will focus on the 80 m^2^ excavated in the small chamber in 1953–1954.

## Stratigraphy

**Fig. 3:**
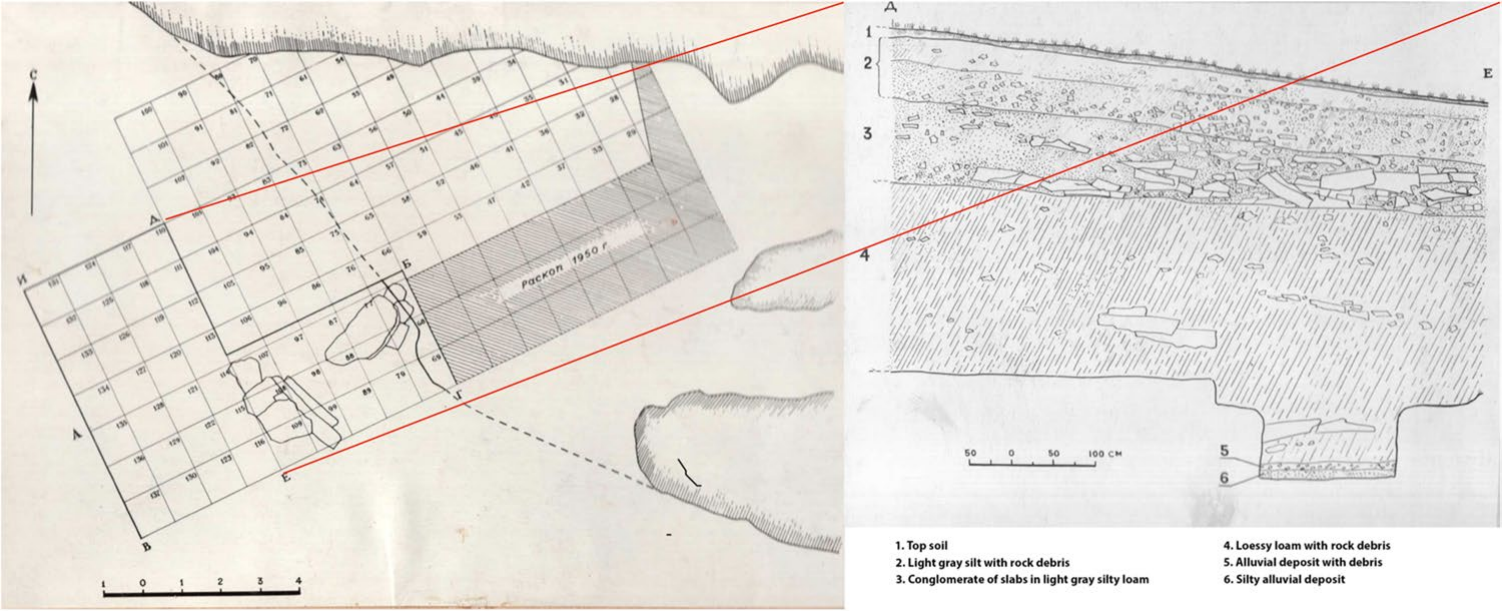
Horizontal plan of the excavation with an annotated transversal section from squares Д-E (courtesy of the East Kazakhstan Regional Museum of Local History, Öskemen, Kazakhstan). Translation: RI

Based on the profile drawings and the descriptions of the layers and their thicknesses, the deepest part of the excavation, in the southwestern corner corresponding to the 1953 pit, reached 3 m, whereas the shallowest was only 0.3 (at the entrance). The sequence is as follows (see Fig. 3):

1. modern debris and soil covered the first 0.25–0.3 m;
2. this was followed in some places by brownish sandy loam;
3. and, finally, by yellow loam.

The sequence ended with two layers of river gravels. The first three layers contained all the finds, and it appears that the brownish layer only filled in the floor cavities. According to the report, the excavators were not able to distinguish cultural levels. Given the nature of the fauna, which suggests carnivore activity in the earliest part of the sequence (see the “Faunal Analysis” section), it is reasonable to assume that some of the lateral unconformities are possibly the result of carnivore den management.

Nevertheless, the excavators noted some features, such as a hearth, and the presence of fire is also attested by five burnt bones.

## Lithic Assemblage

Gokhman reported a very small lithic assemblage, with a total of ca. 50 pieces recovered from the entire excavated surface over several seasons. The drawings and description are very general, but hint at the presence of Levallois and blade technology, concluding that the assemblage is probably Upper Paleolithic. We initially believed the lithic collection to have been shipped to St. Petersburg like the faunal collection, with only a few pieces left behind to feature in the vitrine in the museum at Öskemen. However, the collection in Öskemen contains 43 pieces (including those on display), and no lithic collection could be found in any of the St. Petersburg museums (Kunstkamera, Institute for the History of Material Culture). Therefore, we surmise that the entire collection probably remained in Kazakhstan.

**Fig. 4:**
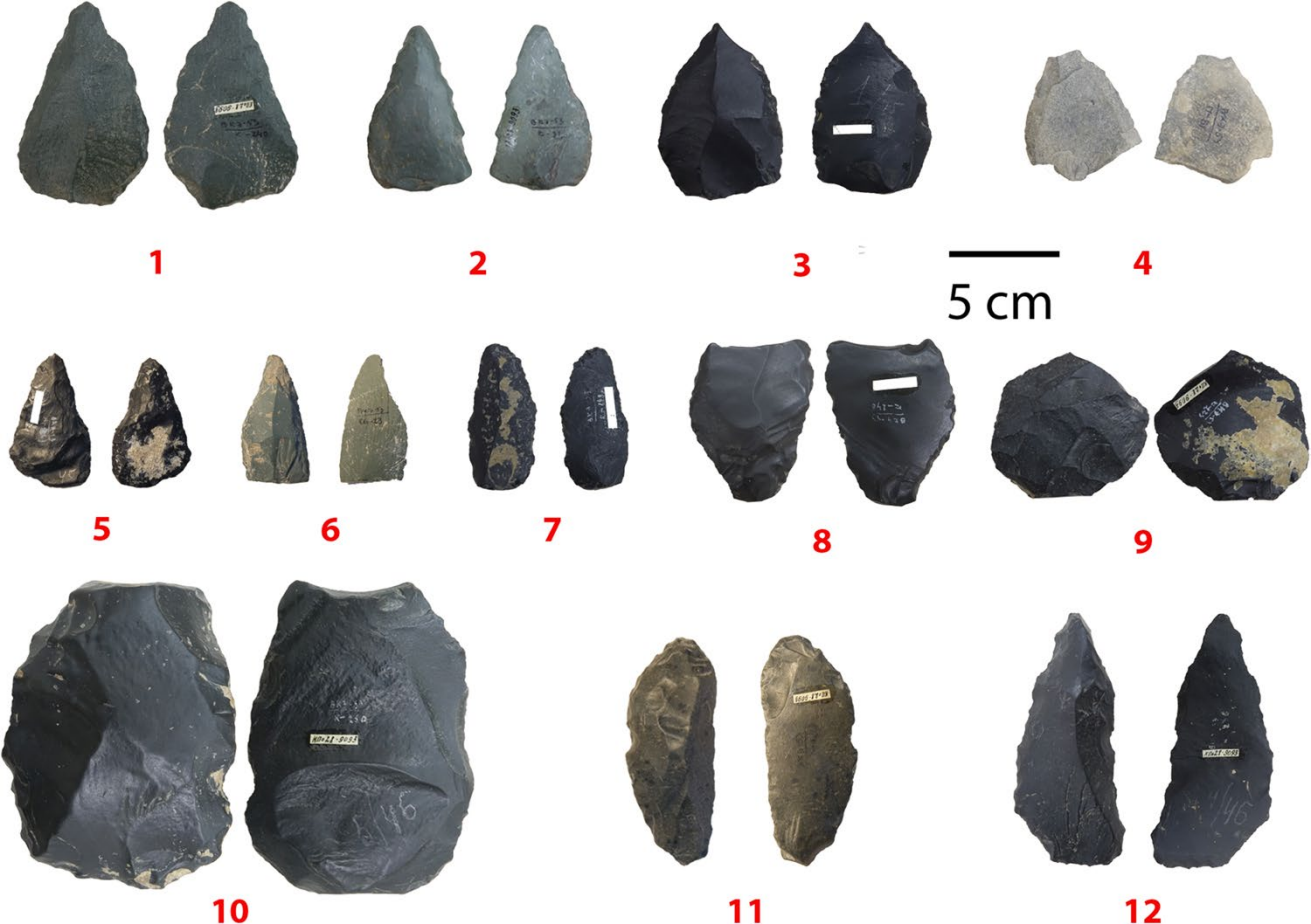
Lithics likely representing the earlier part of the sequence. (1) Scraper with ventral retouch on Levallois flake; (2) retouched Levallois point; (3) double sidescraper on Levallois blank with secondary retouch on the ventral side; (4) Levallois point; (5) flake with bifacial retouch (pointed part is on the retouched bulb of percussion); (6) convergent scraper; (7) convergent scraper with secondary retouch on the ventral aside; (8) proximal double scraper on Levallois blade with secondary ventral retouch; (9) atypical (thick) Levallois flake with ventral retouch; (10) denticulate on atypical Levallois blank; (11) scraper on blade with bifacial retouch at the distal end; (12) convergent scraper on blade. Note the fresher secondary retouch on pieces nos. 1, 3, 11

**Fig. 5:**
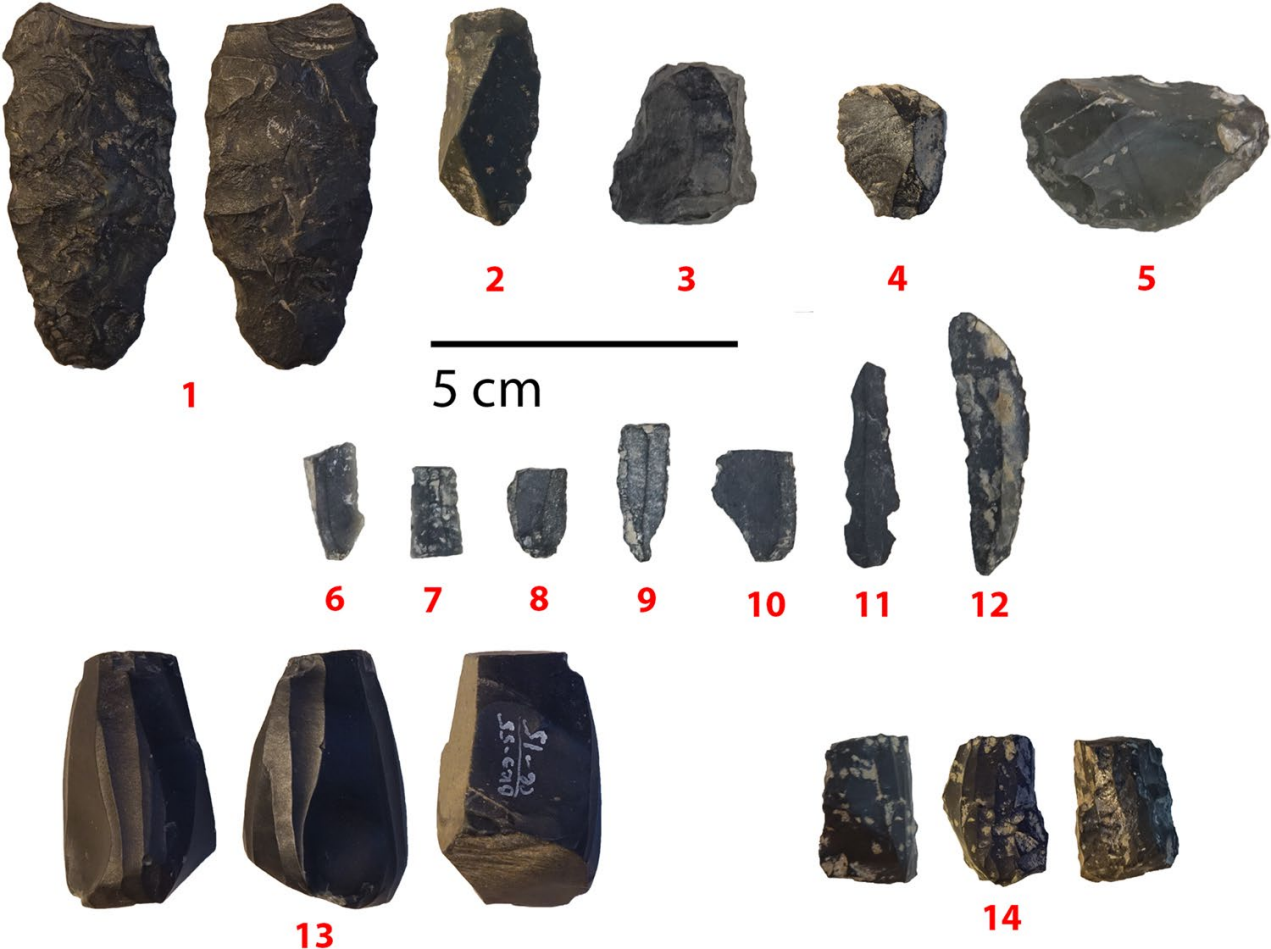
Lithics likely representing the later part of the sequence. (1) Bifacial tool; (2–5) scrapers; (6–12) bladelets; (13–14) bladelet cores

The artifacts obviously belong to different periods, with several pieces typical for an earlier period such as the Middle Paleolithic (see Fig. 4) and others certain to belong to a much later period, probably Holocene (Fig. 5). The earlier period is represented by seven pieces with complex scar patterns and/or facetted platforms typical of the Levallois technique, as well as sidescrapers and bifacially retouched pieces. Four blades and seven bladelets and one microblade core represent a likely Holocene part of the assemblage. Artifacts that could be from the Upper Paleolithic, such as the three endscrapers and four blades, are more difficult to place, since they occur in both Paleolithic and Holocene contexts (Figs. 4 and 5).

**Fig. 6:**
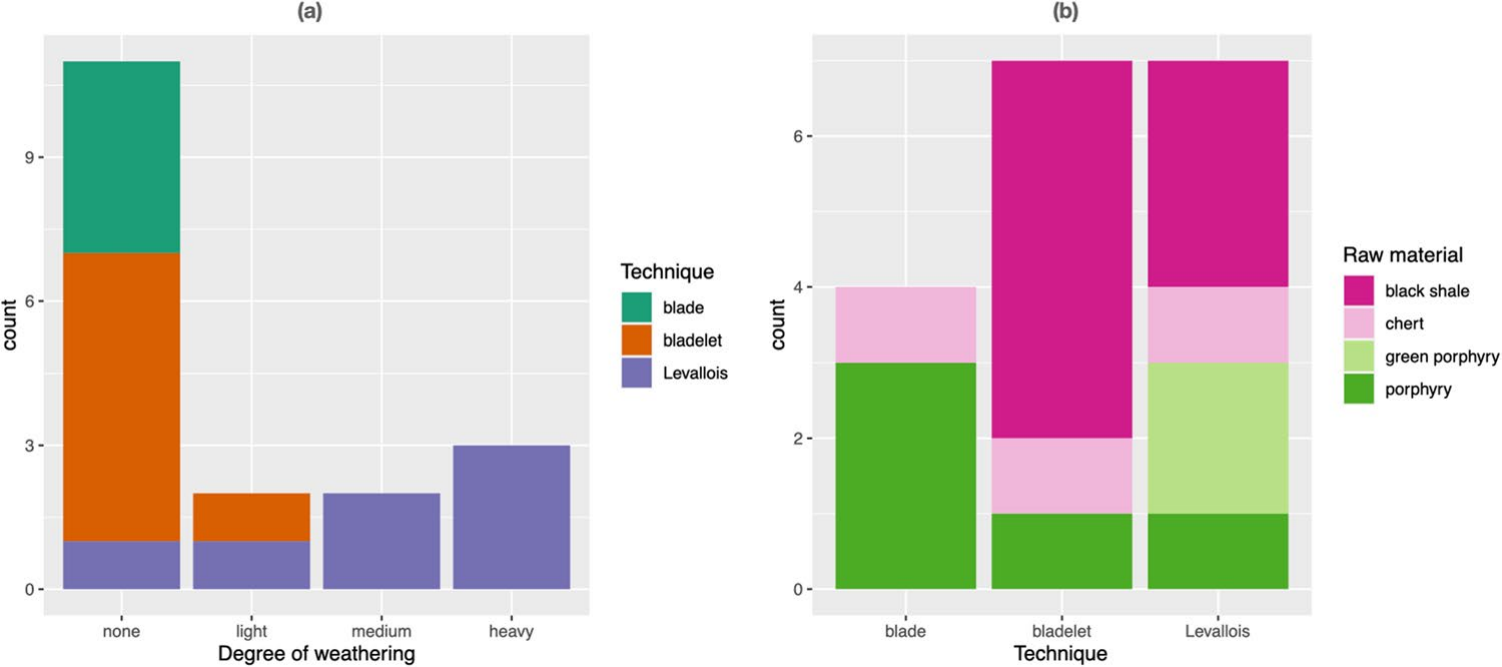
Association between knapping technique and (**a**) degree of weathering and (**b**) type of raw material

The pieces are made on a variety of raw materials, including shale, chert, and porphyry, which are common in the region. Several of the Levallois pieces are made on green porphyry, the only observed association between a raw material and a technique (see Fig. 6). The pieces have distinct degrees of weathering, from very heavily smoothed out (ridges are almost invisible, see Fig. 4, nos. 2, 8, 10, 11), to completely fresh (e.g., Fig. 4, nos. 4 and 6 and Fig. 5). The fact that Gokhman notes the absence of patina or weathering (1957:57) is a further indication of the presence of some water channels that may have affected some parts of the cave but not others.

Although the sample is small, the heaviest weathering is associated with the Levallois pieces (see Fig. 6a, chi-square = 12.623, df = 6, *p*-value = 0.04942). These pieces were often secondarily retouched (e.g., three inverse sidescrapers), probably at a later time, and possibly by different inhabitants of the cave.

## Faunal Analysis

Our analysis was conducted in January 2020 at the Zoological Institute of the Russian Academy of Sciences in St Petersburg, Russia. The faunal remains were previously identified by Vereschchagin and Mel’nikova (1958) and sorted by taxa. The remains of 29 species of birds were studied by Panteleev (2015), but these were not in the collection we accessed in 2020. A second anatomical identification of the mammalian remains was performed during this analysis. Non-identified remains were classified following ungulate size classes (Brain, 1981). With regard to the skeletal part profiles, all identifiable specimens (including shaft fragments) were taken into account and recorded following the “element, portion, segment” method (Gifford & Crader, 1977). Analyses of the bone surfaces were conducted on all the remains. Bone surfaces were observed for the taphonomic and zooarchaeological observations under a low-angled light using a hand lens with magnification 20 × . Weathering, root etching, anthropogenic, and carnivore modifications were systematically investigated (Behrensmeyer, 1978; Binford, 1981; Blumenschine et al., 1996; D’Errico & Villa, 1997; Olsen & Shipman, 1988; Pickering & Egeland, 2006). Oxide colorations of the bone cortical surfaces were also recorded. The proportion of preserved cortical surface was estimated per quartile (Rendu, 2010) to estimate the proportion of anthropogenic modifications. Three remains were selected for ZooMS analysis. We sampled following the Brown et al. (2016) protocols and the analysis was conducted within the ZooSCAn ZooMS platform in Novosibirsk.

### Results of the Zooarchaeological Analysis

The mammalian faunal assemblage contains 279 remains, of which 229 could be taxonomically identified. The high identification rate can be explained by the selective sampling of the assemblage during excavation. This selection is underlined by the abundance of teeth (NISP = 50; %NISP = 18%) and the over-representation of articular extremities for the long bone (NISP = 56; %NISP long bones = 60%), a pattern that is very different from what is usually identified in unselected assemblages from Middle Paleolithic (Costamagno et al., 2006; Daujeard et al., 2012; Stiner, 2013) or Upper Paleolithic (Rendu et al., 2019; Soulier, 2013) contexts accumulated by either humans or carnivores. Herbivores largely dominate the faunal spectra (NISP = 183; %NISP = 80%). Represented by the most common species of the late Pleistocene in the region (Vasiliev, 2013; Vasiliev et al., 2017), the carnivore guild accounts for 16% of the identified remains. One human metacarpal was also identified.

The Bukhtarma Cave faunal spectrum contains taxa coming from different environments (mammoth step, open steppe, woodland), and different chorological contexts (Pleistocene, Holocene), and includes wild and domesticated species, proving that the assemblage results from different archaeological horizons spanning the Pleistocene and the Holocene (Table 1).

**Table 1:** Faunal spectrum of Bukhtarma Cave expressed in NISP and % NISP

Taxa		NISP	% NISP
Rodent	Marmot	7	3.1%
	Beaver	1	0.4%
Carnivores	Badger	1	0.4%
	Fox	3	1.3%
	Wolf	2	0.9%
	Cave hyena	17	7.4%
	Brown bear	8	3.5%
	Medium-size carnivore	1	0.4%
	Large-size carnivore	5	2.2%
Herbivores	Horse	69	30.1%
	Hemiones	4	1.7%
	Camel	15	6.6%
	Rhinoceros	6	2.6%
	Mammoth	2	0.9%
	Bovine	51	22.3%
	Yak	1	0.4%
	Sheep	8	3.5%
	Giant deer	1	0.4%
	Roe deer	6	2.6%
	Red deer	20	8.7%
Human		1	0.4%
Total identified remains		229	
Non-identified	Small-size ungulate	1	
	Medium-size ungulate	5	
	Large-size ungulate	21	
	Megafauna	19	
	NID	4	
TOTAL		279	

**Fig. 7:**
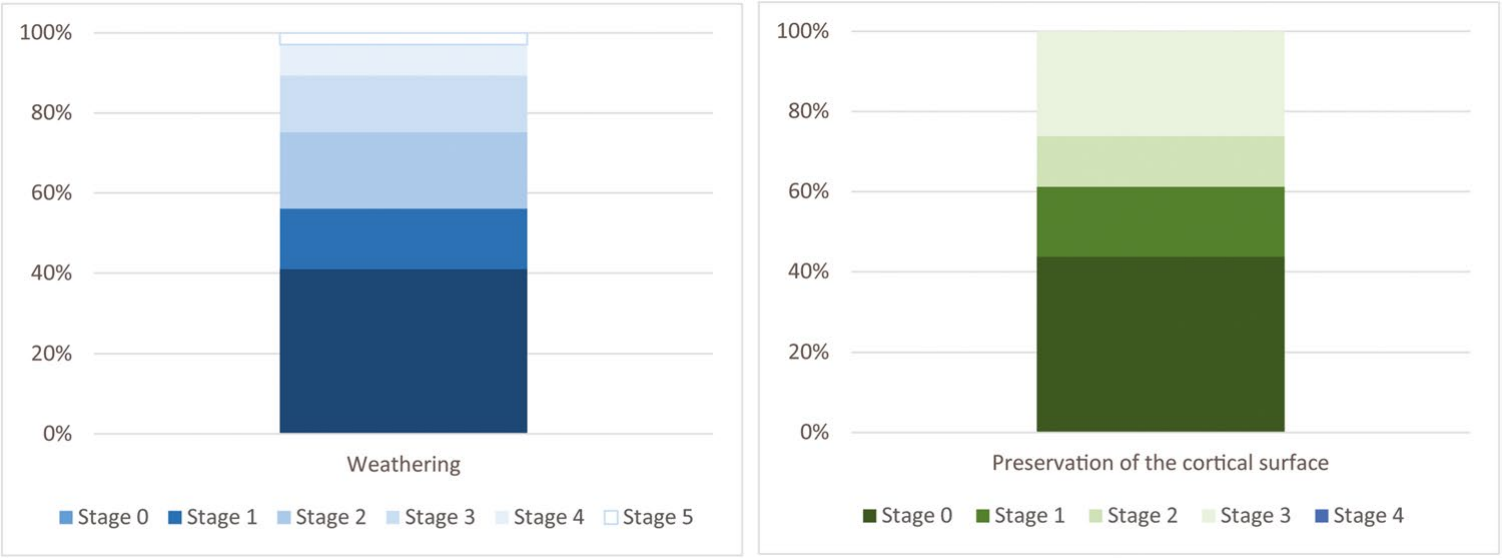
**a** Frequency of the weathering stage on the assemblage; **b** frequency of the cortical surface preservation: stage 1—more than 75% preserved; stage 2—more than 50%; stage 3—more than 25%; stage 4—less than 25%

The preservation of the material is marked by the high frequency (60%) of weathering (Behrensmeyer, 1978), of which the most intense stages (3 to 5) affect 30% of the remains, suggesting that the material was exposed for a long time before burial. These alterations have direct consequences on the preservation of the remains: 40% of them exhibit the destruction of more than half of their cortical surface, limiting the observation of possible human modifications (Fig. 7).

Despite this problem of preservation, carnivore modifications on the assemblage were found in abundance (NISP = 39). The carnivore impact is observable on most of the ungulate species and includes pits, crenulated edges, groove marks, and digested bones (Table 2).

**Table 2:** Carnivore impact on the material compared to the number of observed remains (preservation stages 1 and 2, teeth excluded) and the total of NISP

Taxa	NISP with carnivore mark	NISP total
Brown bear	3	8
Medium-size carnivore	1	1
Large-size carnivore	3	5
Horse	10	69
Hemiones	1	4
Camel	5	15
Mammoth	1	2
Bovine	12	51
Sheep	1	8
Roe deer	1	6
Red deer	1	20
Medium-size ungulate	1	5
Large-size ungulate	3	21
Megafauna	1	5
Non-identified	1	4

**Fig. 8:**
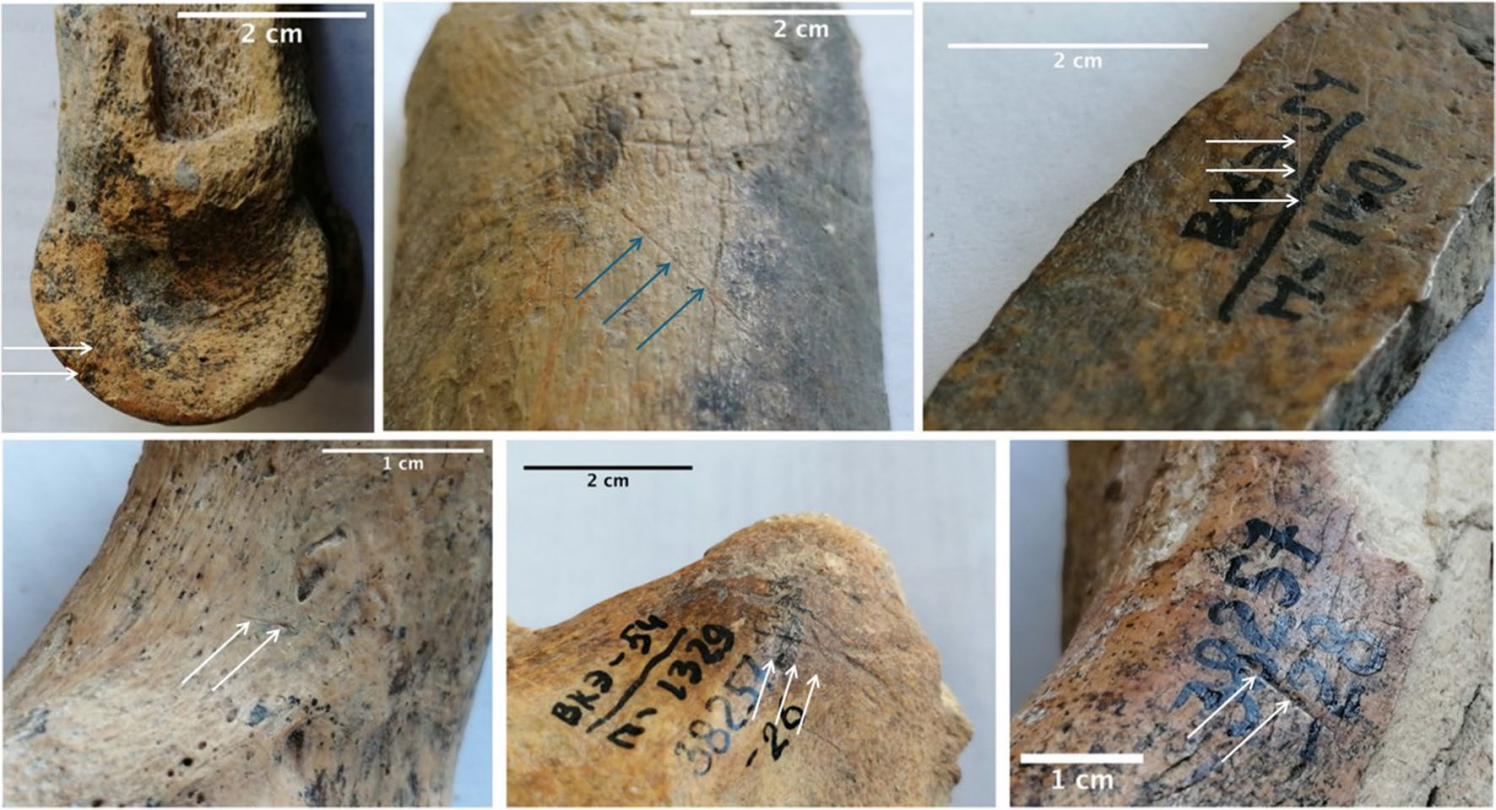
Six examples of cut marked bones from the Bukhtarma Cave collection used for radiocarbon dating. From left to right and top to bottom: *Equus* (MAMS-45706), *Equus* (MAMS-45707), *Ursus* (MAMS-45711), large ungulate (MAMS-45716), *Equus* (MAMS-45705), 6. *Equus* (MAMS-45712)

Fifteen cut marks and six calcined bones make up the human impact on the material (NISP = 21, 18% of the remains with a good preservation of the cortical surfaces (stages 1 and 2)). Human activity was focused on three ungulates species (red deer, bovine, and horse) and on brown bear. The absence of notches and percussion marks can be explained by the scarcity of ungulate long bone shaft fragment (NISP = 45) within the faunal stock (Table 3) (Fig. 8).

**Table 3:** Human impact on the material compare to the number of observed remains (preservation stages 1 and 2, teeth excluded) and the total of NISP

Taxa	NISP with anthropogenic marks	Observable remains	NISP total
Brown bear	2	4	8
Horse	8	29	69
Bovine	2	21	51
Red deer	6	16	20

In addition, five remains of horse (a metacarpal), bison (an atlas, a humerus shaft fragment), and non-identified large ungulates (two long bone shaft fragments) exhibit at the same time carnivore and human marks, suggesting that during some specific moments of the site’s history, the cave was occupied sub-contemporaneously by the two taphonomic agents.

In order to obtain the chronology of the different occupations, 12 remains, including 10 exhibiting anthropogenic and/or carnivore modifications (see Fig. 8), were selected for direct radiocarbon dating, which was carried out at the Curt Engelhorn Zentrum für Archäometrie (CEZA), Mannheim, Germany. The results are presented in Table 4.

**Table 4:**
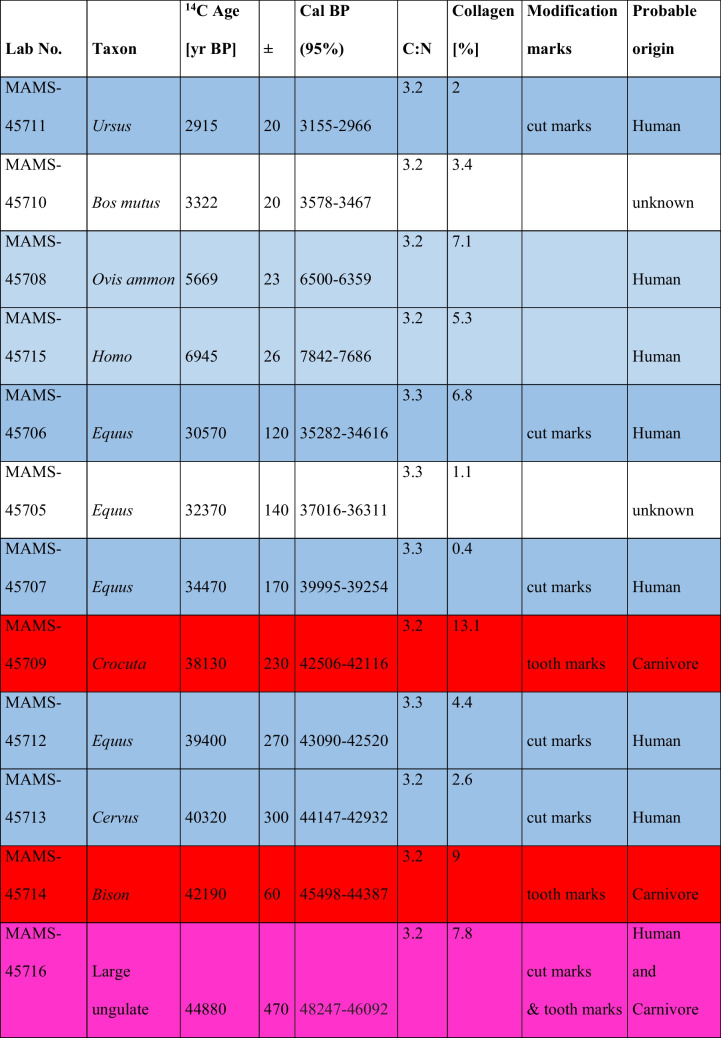
Results of the ^14^C dating of the collection order by chronological order. Dates were calibrated using OxCal 4.4 (Bronk Ramsey, 2021) and the IntCal2020 dataset (Reimer et al., 2020). In blue: remains with a probable human origin; in red: remains with a probable carnivore origin; in purple: remains with a mixed origin

## Discussion

Considering the relative abundance of anthropogenic modifications (cut marks) made by lithics, as well as the abundance of carnivores in the faunal spectrum and the frequency of their marks on the material, it seems that both taphonomic agents (humans and carnivores) contributed to accumulating the assemblages. The dating confirms the observation made on the faunal spectrum and the lithics and the probable existence of multiple occupations of the cave during the Upper Pleistocene. The human occupation dates back to ca. 47 ky cal. BP as attested by the cut mark on a shaft fragment of a large ungulate (#1401). People subsequently returned in the cave at least three other times during the Pleistocene and several times during the Holocene. One of these last occupations is attested by the presence of the 7.7 ky cal. BP old human metacarpal.

We tentatively associate the earliest occupation with the typologically Middle Paleolithic tools found at the site. From that point of view, these are similar to tools known from the Gornyi Altai sites (Derevianko et al., 1998). However, as the majority of these tools appear to have been secondarily recycled at a later (unknown) date, some of their morphological characteristics are mixed and may give the impression of an evolving technology (Coco et al., 2020). The part of the collection that seems, on typological grounds, to belong to an earlier time period is different from assemblages from contemporary nearby sites typically assigned to the Initial Upper Paleolithic (IUP), such as Ushbulaq, a recently excavated open air site in East Kazakhstan (Anoikin et al., 2019; Kharevich et al., 2022). However, at least one bifacially retouched flake shows echoes of possible Eastern Micoquian technology, which is known from Okladnikov and Chagyrskaya Caves in the Gorny Altai, grouped under the Sibiryachikha industry (Derevianko et al., 2013) and linked with a possible long-distance migration of Neanderthal groups from Europe to Siberia (Kolobova et al., 2020).

The intensification of human and carnivore interaction during MIS3 previously described at a local (Vasiliev et al., 2017) and continental scale (Discamps, 2014) finds an interesting echo here. During the first part of the site’s history, between ca. 48 and 40 ky cal. BP, Bukhtarma Cave was occupied alternatively by carnivores and hominins. Rivals et al. (2022) recently concluded that the majority of the Bukhtarma Cave assemblage has no reliable cut marks and represents a carnivore den. We show that this is clearly not the case for the part of the assemblage dated to the Upper Paleolithic. Even for the earliest period, it appears that some of these carnivore occupations took place probably sub-simultaneously to the human occupation since five bones with cut marks were secondarily gnawed on by carnivores before they lost their nutritive value. Finally, the human-carnivore interaction at Bukhtarma Cave ends with the exploitation of bear during the Holocene.

## Conclusions

For now, Bukhtarma Cave remains the only Paleolithic cave site in East Kazakhstan, and possibly the only one to preserve Middle Paleolithic stone tools. Our work gives a first chronological framework establishing the baseline for an occupation of the southern (Kazakh) Altai. The low density of artifacts is likely due to sporadic visits by a small population, interspersed with carnivore occupations, as has also been shown from the Siberian Altai. Over time, after ca. 40 ky, humans become the sole accumulators of the faunal remains. Unfortunately, due to the lack of precise coordinates for the artifacts, the details of human behavior cannot be reconstructed in higher resolution from the collections and the excavation documentation, but Bukhtarma Cave represents an important Late Pleistocene Paleolithic site in Kazakhstan and a reference point for further work in the region.

## Data Availability

All data are publicly available and can be downloaded at 10.5281/zenodo.11966095.

## References

[CR1] Anoikin, A. A., Pavlenok, G. D., Kharevich, V. M., Taimagambetov, Z. K., Shalagina, A. V., Gladyshev, S. A., Ulyanov, V. A., Duvanbekov, R. S., & Shunkov, M. V. (2019). Ushbulak—A new stratified upper Paleolithic site in Northeastern Kazakhstan. *Archaeology, Ethnology & Anthropology of Eurasia,**47*(4), 16–29. 10.17746/1563-0110.2019.47.4.016-029

[CR2] Behrensmeyer, A. K. (1978). Taphonomic and ecologic information from bone weathering. *Paleobiology,**4*(2), 150–162. 10.1017/S0094837300005820

[CR3] Binford, L. R. (1981). *Bones: Ancient Men and Modern Myths*. Academic Press.

[CR4] Blumenschine, R. J., Marean, C. W., & Capaldo, S. D. (1996). Blind tests of inter-analyst correspondence and accuracy in the identification of cut marks, percussion marks, and carnivore tooth marks on bone surfaces. *Journal of Archaeological Science,**23*(4), 493–507. 10.1006/jasc.1996.0047

[CR5] Brain, C. K. (1981). *The hunter or the hunted*?: *An introduction to african cave taphonomy*. University of Chicago Press.

[CR6] Bronk Ramsey, C. (2021). *OxCal v4.4.4*. https://c14.arch.ox.ac.uk/oxcal.html. Accessed 1 Jun 2024.

[CR7] Brown, S., Higham, T., Slon, V., Pääbo, S., Meyer, M., Douka, K., Brock, F., Comeskey, D., Procopio, N., Shunkov, M., Derevianko, A., & Buckley, M. (2016). Identification of a new hominin bone from Denisova Cave, Siberia using collagen fingerprinting and mitochondrial DNA analysis. *Scientific Reports,**6*(1), 23559. 10.1038/srep23559 27020421 10.1038/srep23559 PMC4810434

[CR8] Chernikov, S. S. (1952). Vostochno-Kazakhstanskai͡a Ėkspedit͡sii͡a 1950 g. [The East Kazakhstan Expedition of 1950]. *Kratkie soobshchenii͡a o dokladakh i polevykh issledovanii͡akh Instituta istorii materialʹnoĭ kulʹtury AN SSSR*, *48*, 81–92. http://www.archaeolog.ru/media/books_ksia/ksia_048.pdf

[CR9] Coco, E., Holdaway, S., & Iovita, R. (2020). The effects of secondary recycling on the technological character of lithic assemblages. *Journal of Paleolithic Archaeology*. 10.1007/s41982-020-00055-4

[CR10] Costamagno, S., Liliane, M., Cédric, B., Bernard, V., & Bruno, M. (2006). Les Pradelles (Marillac-le-Franc, France): A mousterian reindeer hunting camp? *Journal of Anthropological Archaeology,**25*(4), 466–484.

[CR11] D’Errico, F., & Villa, P. (1997). Holes and grooves: The contribution of microscopy and taphonomy to the problem of art origins. *Journal of Human Evolution,**33*(1), 1–31. 10.1006/jhev.1997.0141 9236076 10.1006/jhev.1997.0141

[CR12] Daujeard, C., Fernandes, P., Guadelli, J.-L., Moncel, M.-H., Santagata, C., & Raynal, J.-P. (2012). Neanderthal subsistence strategies in Southeastern France between the plains of the Rhone Valley and the mid-mountains of the Massif Central (MIS 7 to MIS 3). *Quaternary International,**252*, 32–47. 10.1016/j.quaint.2011.01.047

[CR13] Derevianko, A. P., Markin, S. V., & Shunkov, M. V. (2013). The Sibiryachikha facies of the Middle Paleolithic of the Altai. *Archaeology, Ethnology and Anthropology of Eurasia,**41*(1), 89–103. 10.1016/j.aeae.2013.07.008

[CR14] Derevianko, A. P., Agadjanian, A. K., Baryshnikov, G. Y., Dergacheva, M., Dupal, T., Malaeva, E., Markin, S. V., Molodin, V., Nikolaev, S., & Orlova, L. (1998). *Arkheologii͡a**, **geologii͡a i paleografii͡a Pleistot͡sena i Golot͡sena Gornogo Altai͡a [Archaeology, geology and palaeogeography of the *Pleistocene* and Holocene of the Mountainous Altai]*. Izdatelstvo Instituta Arkheologii i Ėtnografii Sibirskogo Otdelenii͡a Rossiĭskoĭ Akademii Nauk.

[CR15] Derevianko, A. P., Shunkov, M. V., Anoikin, A. A., Taimagambetov, Z. K., Uliyanov, V. A., Kharevich, V. M., Kozlikin, M. B., Markovskii, G. I., Shalagina, A. V., Pavlenok, G. D., Gladyshev, S. A., Chekha, A. M., & Iskakov, G. T. (2017). Arkheologicheskie raboty v Shiliktinskoi doline na vostoke Kazahstana v 2017 godu [Archaeological works in Shilikti valley in the east of Kazakhstan in 2017]. *Problems of Archaeology, Ethnography, Anthropology of Siberia and Neighboring Territories*, *23*, 93–97. http://paeas.ru/x/ru/2017/2017_093-097.pdf

[CR16] Discamps, E. (2014). Ungulate biomass fluctuations endured by Middle and Early Upper Paleolithic societies (SW France, MIS 5–3): The contributions of modern analogs and cave hyena paleodemography. *Quaternary International,**337*, 64–79. 10.1016/j.quaint.2013.07.046

[CR17] Gifford, D. P., & Crader, D. C. (1977). A computer coding system for archaeological faunal remains. *American Antiquity,**42*(2), 225–238.

[CR18] Gokhman, I. Y. (1957). Paleoliticheskai͡a stoi͡anka «Peshchera» na Bukhtarme [The “Peshchera” Paleolithic site on the Bukhtarma river]. *Kratkie soobshchenii͡a o dokladakh i polevykh issledovanii͡a Instituta istorii materialʹnoĭ kulʹtury AN SSSR*, *67*, 54–58.

[CR19] Iovita, R., Varis, A., Namen, A., Cuthbertson, P., Taimagambetov, Z., & Miller, C. E. (2020). In search of a Paleolithic Silk Road in Kazakhstan. *Quaternary International,**559*, 119–132. 10.1016/j.quaint.2020.02.023

[CR20] Kharevich, V., Kharevich, A., Pavlenok, G., Bocharova, E., Taimagambetov, Z., & Anoikin, A. (2022). Ten millennia without the Levallois technique: Primary knapping methods in Initial Upper Paleolithic industries at the Ushbulak site, eastern Kazakhstan. *Archaeological and Anthropological Sciences,**14*(10), 207. 10.1007/s12520-022-01672-6

[CR21] Kolobova, K. A., Roberts, R. G., Chabai, V. P., Jacobs, Z., Krajcarz, M. T., Shalagina, A. V., Krivoshapkin, A. I., Li, B., Uthmeier, T., Markin, S. V., Morley, M. W., O’Gorman, K., Rudaya, N. A., Talamo, S., Viola, B., & Derevianko, A. P. (2020). Archaeological evidence for two separate dispersals of Neanderthals into southern Siberia. *Proceedings of the National Academy of Sciences,**117*(6), 2879–2885. 10.1073/pnas.1918047117 10.1073/pnas.1918047117 PMC7022189 31988114

[CR22] Krajcarz, M. T., Kot, M., Pavlenok, K., Fedorowicz, S., Krajcarz, M., Lazarev, SYu., Mroczek, P., Radzhabov, A., Shnaider, S., Szymanek, M., & Szymczak, K. (2016). Middle Paleolithic sites of Katta Sai in western Tian Shan piedmont, Central Asiatic loess zone: Geoarchaeological investigation of the site formation and the integrity of the lithic assemblages. *Quaternary International,**399*, 136–150. 10.1016/j.quaint.2015.07.051

[CR23] Krylova, A. A. (1959). Novye paleoliticheskie mestonakhozhdenii͡a v Vostochnom Kazakhstane [New Paleolithic localities in East Kazakhstan]. *Kratkie soobshchenii͡a o dokladakh i polevykh issledovanii͡akh Instituta istorii materialʹnoĭ kulʹtury AN SSSR*, *76*, 28.

[CR24] Kunitake, S., & Taimagambetov, Z. K. (2021). Bladelet industries of the Early Upper Palaeolithic in southern Kazakhstan: A detailed analysis of carinated bladelet cores excavated from the newly discovered Buiryokbastau-Bulak-1 site in the Karatau mountains. *Quaternary International*, S1040618221001579. 10.1016/j.quaint.2021.03.016

[CR25] Olsen, S. L., & Shipman, P. (1988). Surface modification on bone: Trampling versus butchery. *Journal of Archaeological Science,**15*(5), 535–553. 10.1016/0305-4403(88)90081-7

[CR26] Ozherelyev, D. V., Lev, S. Yu., & Stolpnikova, E. M. (2023a). Problemy verkhnego paleolita predgoriĭ severnogo Ti͡anʹ- Shani͡a: noveĭshie otkrytii͡a i dalʹneĭshie perspektivy [Problems of the Upper Paleolithic in the foothills of the Northern Tien Shan: latest discoveries and perspectives]. Vestnik Moskovskogo Universiteta. Serija 23. 10.32521/2074-8132.2023.1.118-128

[CR27] Ozherelyev, D. V., Mamirov, T. B., Sarayev, V. V., & Volovenko, V.D. (2023b). Pervye dannye o zaselenii vnutrigornykh raionov Severnogo Tian-Shanya v paleolite [The first data on the settlement of intramountain regions Northern Tien Shan in the Paleolithic]. *Pervobytnai͡a arkheologii͡a. Zhurnal mezhdist͡siplinarnykh issledovaniĭ *[*Prehistoric Archaeology. Journal of Interdisciplinary Studies*] 32–46. 10.31600/2658-3925-2023-1-32-46

[CR28] Panteleev, A. V. (2015). Nakhodki chetvertichnykh ptit͡s v Kazakhstane [Finds of Quaternary birds in Kazakhstan]. *Russkiĭ Ornitologicheskiĭ Zhurnal,**24*(1173), 2749–2752.

[CR29] Pavlenok, G., Bocharova, E., Gladyshev, S., Ulianov, V., Markovskiy, G., Kharevich, V., Taimagambetov, Z., & Anoikin, A. (2021). The Karasai site: The first stratified Mesolithic assemblage in eastern Kazakhstan. *Archaeological Research in Asia,**25*, 100249. 10.1016/j.ara.2020.100249

[CR30] Pickering, T. R., & Egeland, C. P. (2006). Experimental patterns of hammerstone percussion damage on bones: Implications for inferences of carcass processing by humans. *Journal of Archaeological Science,**33*(4), 459–469. 10.1016/j.jas.2005.09.001

[CR31] Reimer, P. J., Austin, W. E. N., Bard, E., Bayliss, A., Blackwell, P. G., Bronk Ramsey, C., Butzin, M., Cheng, H., Edwards, R. L., Friedrich, M., Grootes, P. M., Guilderson, T. P., Hajdas, I., Heaton, T. J., Hogg, A. G., Hughen, K. A., Kromer, B., Manning, S. W., Muscheler, R., … Talamo, S. (2020). The IntCal20 Northern Hemisphere Radiocarbon Age Calibration Curve (0–55 cal kBP). *Radiocarbon*, *62*(4), 725–757. 10.1017/RDC.2020.41

[CR32] Rendu, W. (2010). Hunting behavior and Neanderthal adaptability in the Late Pleistocene site of Pech-de-l’Azé I. *Journal of Archaeological Science,**37*(8), 1798–1810. 10.1016/j.jas.2010.01.037

[CR33] Rendu, W., Renou, S., Soulier, M.-C., Rigaud, S., Roussel, M., & Soressi, M. (2019). Subsistence strategy changes during the Middle to Upper Paleolithic transition reveals specific adaptations of Human Populations to their environment. *Scientific Reports,**9*(1), 15817. 10.1038/s41598-019-50647-6 31676799 10.1038/s41598-019-50647-6 PMC6825241

[CR34] Rivals, F., Baryshnikov, G. F., Prilepskaya, N. E., & Belyaev, R. I. (2022). Diet and ecological niches of the Late Pleistocene hyenas Crocuta spelaea and C. ultima ussurica based on a study of tooth microwear. *Palaeogeography, Palaeoclimatology, Palaeoecology*, *601*, 111125. 10.1016/j.palaeo.2022.111125

[CR35] Rybin, E. P., Nokhrina, T. I., & Taimagambetov, Zh. K. (2014). Pervai͡a radiouglerodnai͡a data dli͡a paleolita Vostochnogo Kazakhstana: K voprosu o prodolzhitelʹnosti sushchestvovanii͡a levalluazskoĭ konvergentnoĭ tekhnologii na Altae [First radiocarbon date for the Paleolithic of East Kazakhstan: On the question of continuation of Levallois convergent technology in the Altai]. *Problemy Arkheologii, Ėtnografii, Antropologii Sibiri i Sopredelʹnykh Territoriĭ,**20*, 83–86.

[CR36] Soulier, M.-C. (2013). Entre alimentaire et technique: L’exploitation animale aux débuts du paléolithique supérieur: Stratégies de subsistance et chaînes opératoires de traitement du gibier à Isturitz, La Quina aval, Roc-de-Combe et Les Abeilles. Toulouse 2. http://www.theses.fr/2013TOU20026

[CR37] Spassky, G. (1818). Sibirskie drevnosti [Siberian antiquities]. *Sibirskiy Vestnik*, *1*.

[CR38] Stiner, M. C. (2013). An unshakable Middle Paleolithic? Trends versus Conservatism in the Predatory Niche and Their Social Ramifications. *Current Anthropology,**54*(S8), S288–S304. 10.1086/673285

[CR39] Taimagambetov, Zh., & Ozherelyev, D. (2009). *Pozdnepaleoliticheskie pamyatniki Kazakhstana [Late Paleolithic sites of Kazakhstan]*. Kazak Un-Ti.

[CR40] Vasiliev, S. (2013). Large mammal fauna from the Pleistocene deposits of Chagyrskaya Cave northwestern Altai (based on 2007–2011 excavations). *Archaeology, Ethnology and Anthropology of Eurasia,**41*(1), 28–44.

[CR41] Vasiliev, S., Shunkov, M., & Kozlikin, M. (2017). Megafaunal remains from the eastern chamber of Denisova cave and problems of reconstructing the Pleistocene environments in the northwestern Altai. *Problems of Archaeology, Ethnography and Anthropology of Siberia and Neighbouring Territories,**23*, 60–64.

[CR42] Vereshchagin, N. K., & Mel’nikova, N. N. (1958). Zoogeograficheskie otkrytii͡a arkheologov v Vostochnom Kazakhstane i v Altaĭskom krae [Zoogeographic discoveries of archaeologists in eastern Kazakhstan and in the Altai Territory]. *Izvestii͡a Vsesoi͡uznogo Geograficheskogo Obshchestva*, *90*(4), 385387.

